# Quantitative bile and serum proteomics for the screening and differential diagnosis of primary sclerosing cholangitis

**DOI:** 10.1371/journal.pone.0272810

**Published:** 2022-08-25

**Authors:** Matilda Holm, Sakari Joenväärä, Mayank Saraswat, Tiialotta Tohmola, Toni Saarela, Andrea Tenca, Johanna Arola, Risto Renkonen, Martti Färkkilä

**Affiliations:** 1 Transplantation Laboratory, Haartman Institute, University of Helsinki and Helsinki University Hospital, Helsinki, Finland; 2 HUSLAB, Helsinki University Hospital, Helsinki, Finland; 3 Department of Laboratory Medicine and Pathology, Mayo Clinic, Rochester, Minnesota, United States of America; 4 Department of Psychology and Logopedics, Faculty of Medicine, University of Helsinki and Helsinki University Hospital, Helsinki, Finland; 5 Clinic of Gastroenterology, University of Helsinki and Helsinki University Hospital, Helsinki, Finland; NIH, UNITED STATES

## Abstract

**Background:**

Primary sclerosing cholangitis (PSC) is a chronic liver disease characterized by biliary strictures, cholestasis, and a markedly increased risk of cholangiocarcinoma. New markers for the screening and differential diagnosis of PSC are needed. In this pilot study, we have analyzed both the bile and serum proteomic profiles of 80 PSC patients and non-PSC controls (n = 6 for bile and n = 18 for serum).

**Aim:**

The aim of this study was to discover candidates for new biomarkers for the differential diagnosis of PSC.

**Methods:**

Bile and serum samples were processed and subsequently analyzed using ultra performance liquid chromatography-ultra definition mass spectrometry (UPLC-UDMS^E^). Further analysis included statistical analyses such as receiver operating characteristic curve analysis as well as pathway analysis using Ingenuity Pathway Analysis.

**Results and conclusions:**

In bile, we discovered 64 proteins with significantly different levels between the groups, with fold changes of up to 129. In serum, we discovered 112 proteins with significantly different levels. Receiver operating characteristic curve analysis found multiple proteins with high area under the curve values, up to 0.942, indicating that these serum proteins are of value as new non-invasive classifiers of PSC. Pathway analysis revealed multiple canonical pathways that were enriched in the dataset, which have roles in bile homeostasis and metabolism. We present several serum proteins that could serve as new blood-based markers for the diagnosis of PSC after further validation. The measurement of serum levels of these proteins could be of use in the screening of patients with suspected PSC.

## Introduction

Primary sclerosing cholangitis (PSC) is a chronic inflammatory disease of the biliary epithelium that leads to strictures of intra- and extrahepatic bile ducts, causing cholestasis and biliary cirrhosis [1]. PSC is associated with a significantly increased risk of cholangiocarcinoma (CCA) [2, 3]. In most patients, disease progression and end-stage liver disease are inevitable and liver transplantation is the only curative option available. While PSC is a rare disease, the incidence is highest in northern Europe, with a prevalence of up to 31.7 per 100 000 individuals [1, 4, 5]. PSC is closely associated with inflammatory bowel disease (IBD), which is present in approximately 70% of PSC patients. PSC patients are also at a 10-fold greater risk of developing colorectal cancer, which can develop at an early age and warrants surveillance from the time of PSC diagnosis [6, 7].

Many PSC patients are asymptomatic before the development of complications, and PSC is usually diagnosed in a work-up of persistently abnormal liver function tests [8, 9]. The diagnosis of PSC is based on cholestatic liver function tests and cholangiography demonstrating the characteristic bile duct changes of PSC. Elevated alkaline phosphatase (ALP) levels can usually be seen in blood tests from PSC patients [5, 8], but levels may fluctuate in PSC patients, and a significant proportion of PSC patients present with normal ALP levels, meaning that normal ALP levels do not exclude the diagnosis of PSC [1, 9]. Unlike for primary biliary cirrhosis, where mitochondrial antibodies serve as a screening test, there is currently a lack of screening tests for PSC. While PSC is an idiopathic disease, multiple conditions can lead to secondary sclerosing cholangitis (SSC), a disease similar to PSC but that originates from a known pathological process. The features of SSC can mimic PSC and distinguishing the two diseases can be challenging [9, 10]. The differential diagnosis between PSC and other cholestatic liver diseases also presents a challenge when selecting patients to undergo invasive investigations such as endoscopic retrograde cholangiography (ERC). New biomarkers for both the screening and differential diagnosis of PSC are therefore needed.

Previous studies have shown that bile proteomics can be used to distinguish PSC from CCA [11, 12]. A proteomic study of urine identified a noninvasive peptide marker model that was capable of differentiating CCA from PSC and benign biliary disorders, although this model was unable to distinguish between patients with PSC and benign biliary disorders [13]. However, there is a paucity of studies comparing the proteomic profiles of non-PSC controls and PSC patients. A study by Vesterhus et al. used a bead-based array that targeted 63 selected proteins to investigate the levels of these proteins in bile samples from PSC patients and controls. The authors selected several proteins for further investigation and measured their levels in serum samples from PSC patients and controls. They found that serum levels of interleukin-8 were associated with disease severity and transplant-free survival but did not propose any serum-based markers for the diagnosis of PSC [14]. The aim of this study was to analyze and compare the bile and serum proteomic profiles of PSC patients and non-PSC controls to discover proteins that could be of value as new, minimally invasive markers for the screening and diagnosis of PSC. We subsequently identified several serum proteins that could serve as new non-invasive blood-based markers for the diagnosis of PSC, although further validation is needed.

## Materials and methods

### Patient samples

This study was approved by the Helsinki University Hospital Ethical Committee IV (HUS/1556/2020) and was carried out in accordance with the principles presented in the Declaration of Helsinki. Written consent was obtained from all patients prior to inclusion in the study. This study analyzed bile and serum samples from 80 patients with confirmed PSC but no dysplasia or CCA as well as 18 serum samples and six bile samples from non-PSC patients in whom PSC was excluded by magnetic resonance cholangiography, ERC, and liver histology and who subsequently served as non-PSC controls. These patients were not diagnosed with PSC after ERC and follow-up for two years. The patients were selected from the prospectively collected PSC registry at Helsinki University Hospital, all of whom had undergone one or more ERCs. The patients were referred for ERC due to suspected PSC. Serum samples were collected at the time of ERC. During ERC, bile samples were aspirated using a balloon catheter and stored in -80°C until used. Brush cytology samples were collected from both extra- and intrahepatic bile ducts for Papanicolaou staining, which was used to grade inflammation and dysplasia. The findings of ERC were scored according to the Helsinki score (modified Amsterdam score, range 2–16) [15]. Detailed patient characteristics are given in S1 Table.

### Sample processing and digestion

#### Bile samples

Bile samples were diluted with 1:1 v/v standard phosphate-buffered saline, pH 7.4. Top 12 protein depletion was performed using Pierce™ Top 12 Abundant Protein Depletion Spin Columns (Pierce, ThermoFisher, MA, USA) according to the manufacturer’s instructions. The total protein concentration after depletion was determined using the Pierce BCA assay kit (Pierce, ThermoFisher, MA, USA) according to the manufacturer´s instructions. Equal amounts (100 μg) of protein from each sample were precipitated using acetone, as follows. Cold acetone (-20°C) was added at a ratio of 4:1 v/v to the samples, which were then briefly vortexed. The samples were incubated in -20°C for 60 minutes, after which they were pelleted by centrifugation for 10 minutes at 16,000 x g and +4°C. The supernatant was removed and the remaining acetone evaporated using a Savant SpeedVac (ThermoFisher, MA, USA). The pellets were then reconstituted in 8M urea. Dithiothreitol (DTT) was added to a final concentration of 5mM and the samples were incubated 30 minutes at 65°C in a thermomixer. Samples were then cooled and iodoacetamide was added to a final concentration of 15mM, after which samples were incubated 30 minutes at 25°C with shaking. DTT was added to a final concentration of 15mM to quench the remaining iodoacetamide and prevent overalkylation. Samples were diluted with 50mM ammonium bicarbonate for a final urea concentration of 1M. One μg of Trypsin Gold (Promega, Southampton, UK) was added to each sample and the mixture was incubated at 37°C overnight. The next day, the samples were cleaned using C18 spin columns (Pierce, Thermo Fisher Scientific) according to the manufacturer’s protocol and and dissolved in 0.1% formic acid with 0.3% acetonitrile and 12.5 fmol/μl of Hi3 spike-in standard peptides (Waters, MA, USA).

#### Serum samples

The serum samples were processed as previously described [16, 17]. Briefly, the serum samples were thawed and top 12 protein depletion was performed using Pierce™ Top 12 Abundant Protein Depletion Spin Columns (Pierce, ThermoFisher, MA, USA) according to the manufacturer’s instructions. The total protein concentration was determined using the Pierce BCA assay kit (Pierce, ThermoFisher, MA, USA) and the amount of serum corresponding to 100 μg protein was aliquoted and dried. The samples were then dissolved in Tris buffer containing urea, after which DTT was added and the samples were shaken for 1 hour at room temperature before iodoacetamide was added. The samples were shaken another hour, after which DTT was added again, and the samples were shaken once more before being diluted with mQ water. Trypsin was added at a ratio of 1:50 trypsin to protein and the samples were digested at 37°C overnight. The following day, 30 μg of tryptic peptides were cleaned using C18 spin columns and dissolved in 0.1% formic acid with 0.3% acetonitrile and 12.5 fmol/μl of Hi3 spike-in standard peptides (Waters, MA, USA).

### Ultra performance liquid chromatography-ultra definition mass spectrometry and quantification

#### UPLC-UDMS^E^

UPLC-UDMS^E^ was performed as previously described [16, 17]. In summary, 4 μl of each sample (around 1.4 μg protein) was injected into a nanoACQUITY UPLC-system (Waters Corporation, MA, USA). For separation, TRIZAIC nanoTile 85 μm x 100 mm HSS-T3u wTRAP was used. The samples were loaded, trapped and washed for two minutes with 8.0 μL/min 1% B (0.1% formic acid in acetonitrile). The analytical gradient used was 0–1 min 1% B, at 2 min 5% B, at 65 min 30% B, at 78 min 50% B, at 80 min 85% B, at 83 min 85% B, at 84 min 1% B and at 90 min 1% B with 450 nL/min. Buffer A was 0.1% formic acid in water. Data were acquired in data-independent acquisition fashion with UDMSE mode using a Synapt G2-Si mass spectrometer (Waters Corporation, MA, USA). The collected data range was 100–2000 m/z, scan time one‐second, IMS wave velocity 650 m/s, and collision energy was ramped in trap between 20 and 60 V. Calibration was performed using Glu1‐Fibrinopeptide B MS2 fragments and Glu1‐Fibrinopeptide B precursor ion was used as a lock mass during the runs. All samples were run in triplicates and the coefficient of variation for the dataset was 4.1%.

#### Data analysis

Data analysis and label-free quantification were performed as previously described, using Progenesis QI for Proteomics (Nonlinear Dynamics, Waters Corporation, MA, USA). This software uses ProteinLynx Global Server (Waters Corporation, MA, USA) as a protein identification search engine [16–18]. The peptide identification was done against Uniprot human FASTA sequences (release 2018_10). Default peptide and fragment error tolerances were used. The false-discovery rate was set to maximally 1%, after which 301 bile proteins with two or more unique peptides and 207 serum proteins with two or more unique peptides were identified. The parsimony principle was used to group the proteins, but due to over-stringency, Progenesis QI for proteomics does not follow a strict parsimonious approach [19]. Therefore, if two proteins are found with common peptides, the protein with fewer peptides is absorbed into the protein with more peptides. Relevant proteins are listed as a group under the lead protein with the highest coverage or score. Quantitation is performed with the lead identity peptide data. All peptides unique to the protein sequence were used for quantification. Further details are available on Nonlinear Dynamics’ website (www.nonlinear.com). The mass spectrometry proteomics data generated in this project have been deposited to the ProteomeXchange Consortium via the PRIDE [20, 21] partner repository with the dataset identifier PXD026694.

### Further analysis

The differences between the two groups (PSC vs. controls) were analyzed using the Mann-Whitney U test. Pathway analysis was performed using Ingenuity Pathway Analysis (IPA) (QIAGEN Bioinformatics, Redwood City, CA). Only proteins that passed the cutoff of a Mann-Whitney p-value of less than 0.05 were used for pathway analysis. Receiver operating characteristic (ROC) curve analysis was performed and ROC curves created using R (https://www.r-project.org). The results are reported as area under the curve (AUC) values.

## Results

### Bile proteomics of PSC patients and controls

#### Protein identification

A total of 301 proteins with two or more unique peptides, given in S2 Table, were quantified. These proteins were subsequently used for further analysis.

#### Differentially expressed proteins

When bile samples from PSC patients and non-PSC controls were compared, 64 proteins passed the requirement of having a Mann-Whitney U test p-value of less than 0.05. These 64 proteins were considered to have significantly different levels between the groups. The top 20 proteins according to fold change are given in Table 1 and all 64 proteins can be found marked in blue in S2 Table. The protein with the largest fold change was ezrin, which displayed significantly higher levels in PSC patients and had a fold change of 129 compared to non-PSC controls. Multiple other proteins also displayed very large fold changes between the two groups, such as protein S100-P, with a fold change of 76.7, mucin-like protein 1, with a fold change of 55, and apolipoprotein E, with a fold change of 26.1. All but two significantly different proteins had higher levels in the bile of PSC patients as compared to the non-PSC controls.

**Table 1:** The top 20 proteins according to fold change that passed the cutoff of a Mann-Whitney p-value of less than 0.05 when bile samples from PSC patients and non-PSC controls were compared.

Accession	Peptide count	Unique peptides	Confidence score	Mann-Whitney P-value	Max fold change (PSC/control)	Power	Protein name	Gene name
P15311	2	2	9,8	8,02E-03	129,0	0,996	Ezrin	EZR
P25815	5	4	51,1	7,78E-03	76,7	0,995	Protein S100-P	S100P
Q96DR8	2	2	8,8	7,48E-03	55,0	0,995	Mucin-like protein 1	MUCL1
P02649	11	9	74,4	4,19E-03	26,1	0,987	Apolipoprotein E	APOE
P26447	3	2	22,1	1,92E-03	21,9	1,000	Protein S100-A4	S100A4
P80723	7	6	48,6	4,58E-02	19,1	0,935	Brain acid soluble protein 1	BASP1
Q6P4A8	4	3	26,6	1,41E-02	17,7	0,786	Phospholipase B-like 1	PLBD1
P63104	3	2	19,7	1,59E-02	17,4	0,998	14-3-3 protein zeta/delta	YWHAZ
Q8IXQ9	3	2	12,3	1,74E-02	14,8	0,895	Electron transfer flavoprotein beta subunit lysine methyltransferase	ETFBKMT
P12838	3	2	14,6	3,29E-02	13,5	0,876	Neutrophil defensin 4	DEFA4
P31949	7	5	62,0	1,82E-02	13,0	0,984	Protein S100-A11	S100A11
P80511	6	6	38,7	7,50E-03	12,8	1,000	Protein S100-A12	S100A12
P06748	2	2	17,0	1,65E-02	11,4	0,999	Nucleophosmin	NPM1
P56470	3	2	17,0	2,14E-03	10,7	0,999	Galectin-4	LGALS4
P46940	4	3	23,0	2,14E-02	10,5	0,955	Ras GTPase-activating-like protein IQGAP1	IQGAP1
P59665;P59666	22	16	82,5	1,47E-02	9,4	0,993	Neutrophil defensin 1	DEFA1
O75594	3	3	24,8	5,91E-03	8,8	0,996	Peptidoglycan recognition protein 1	PGLYRP1
Q9HD89	7	6	55,9	1,08E-02	8,3	0,986	Resistin	RETN
P19013	15	4	131,1	4,83E-02	7,5	0,660	Keratin_ type II cytoskeletal 4	KRT4
P02042	41	28	202,7	1,13E-02	6,5	0,966	Hemoglobin subunit delta	HBD

#### Pathway analysis

**Fig 1:**
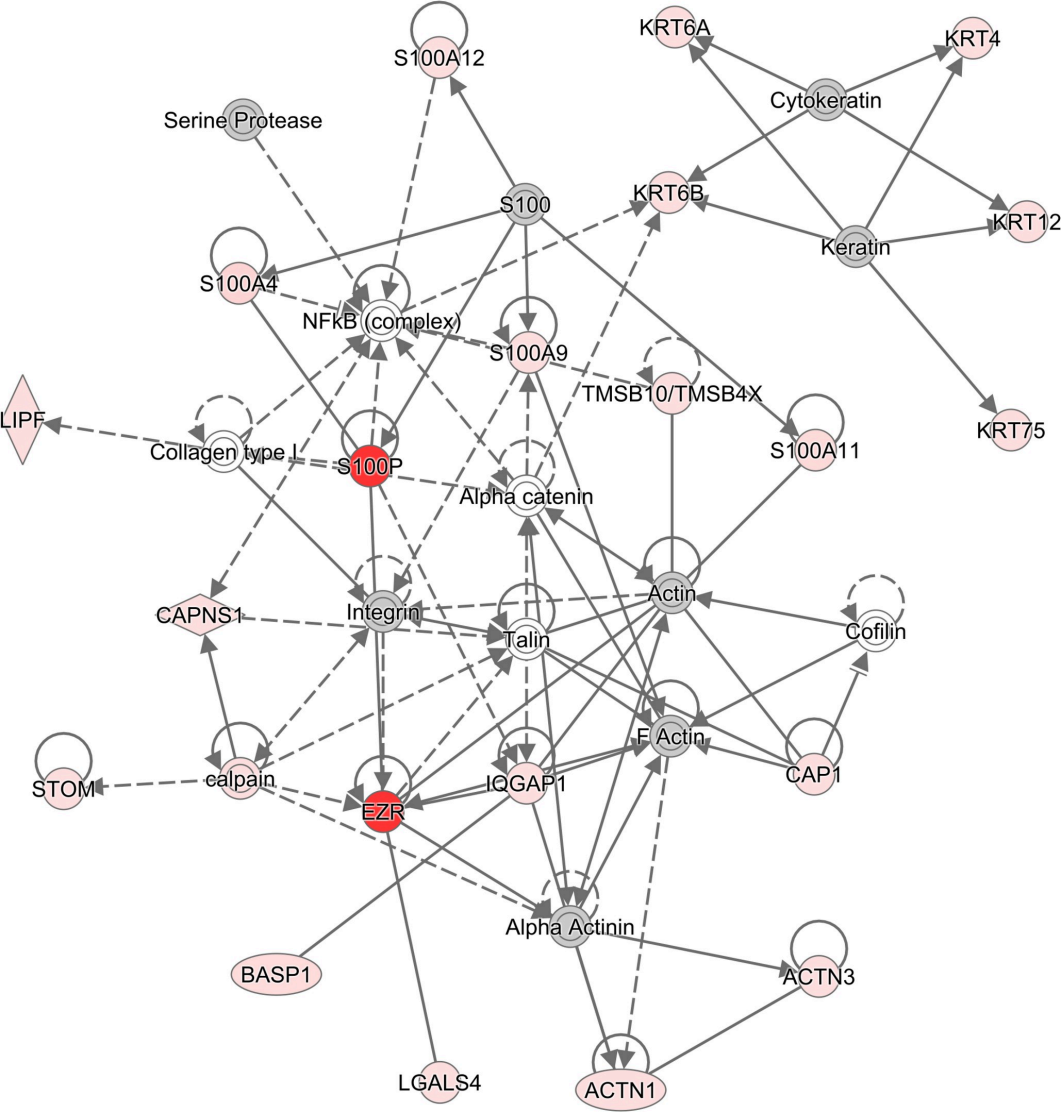
The top network of protein-protein interactions generated by IPA when bile samples from PSC patients and controls were compared. Proteins in red are proteins that were identified in this study and which displayed higher levels in PSC patients. Proteins in gray are proteins that were identified in this study and that were neither significantly up- nor downregulated. Proteins in white are proteins that, although not identified in this study, play roles in this network and interact with the proteins identified.

Pathway analysis was also performed using IPA and discovered multiple canonical pathways that were enriched in the bile protein dataset. The top five pathways enriched were Production of Nitric Oxide and Reactive Oxygen Species in Macrophages, liver X receptor (LXR)/retinoid X receptor (RXR) Activation, Regulation of Cellular Mechanics by Calpain Protease, Atherosclerosis Signaling, and farnesoid X receptor (FXR)/RXR Activation. All canonical pathways enriched are given in S1 Fig. The top network of protein-protein interactions also generated by IPA is shown in Fig 1 and was found to be associated with the following functions: Cell Morphology, Embryonic Development, and Hair and Skin Development and Function. Only proteins that passed the cutoff of a Mann-Whitney p-value of less than 0.05 were used for pathway analysis.

### Serum proteomics of PSC patients and controls

#### Protein identification

Serum samples from the same 80 PSC patients from whom bile samples were previously analyzed and 18 controls were analyzed. One serum sample (marked in S1 Table) from a PSC patient did not normalize and was subsequently excluded from further analysis, leaving a total of 79 serum samples. A total of 207 proteins with two or more unique peptides were quantified and are listed in S3 Table.

#### Differentially expressed proteins

**Fig 2:**
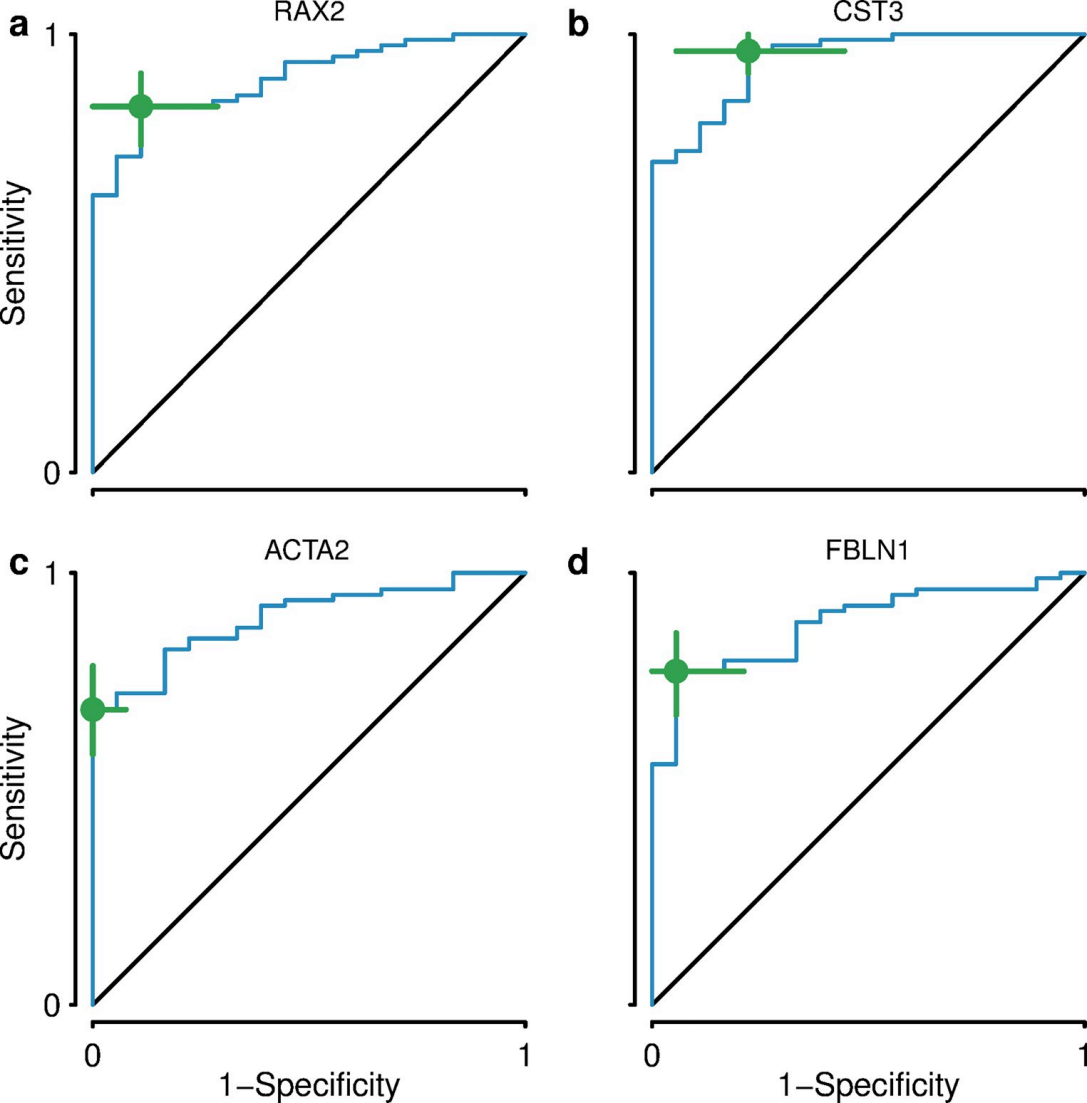
The ROC curves for the four serum proteins with the highest AUC values. A) The ROC curve for retina and anterior neural fold homeobox protein 2. B) The ROC curve for cystatin-C. C) The ROC curve for actin, aortic smooth muscle. D) The ROC curve for fibulin-1.

**Fig 3:**
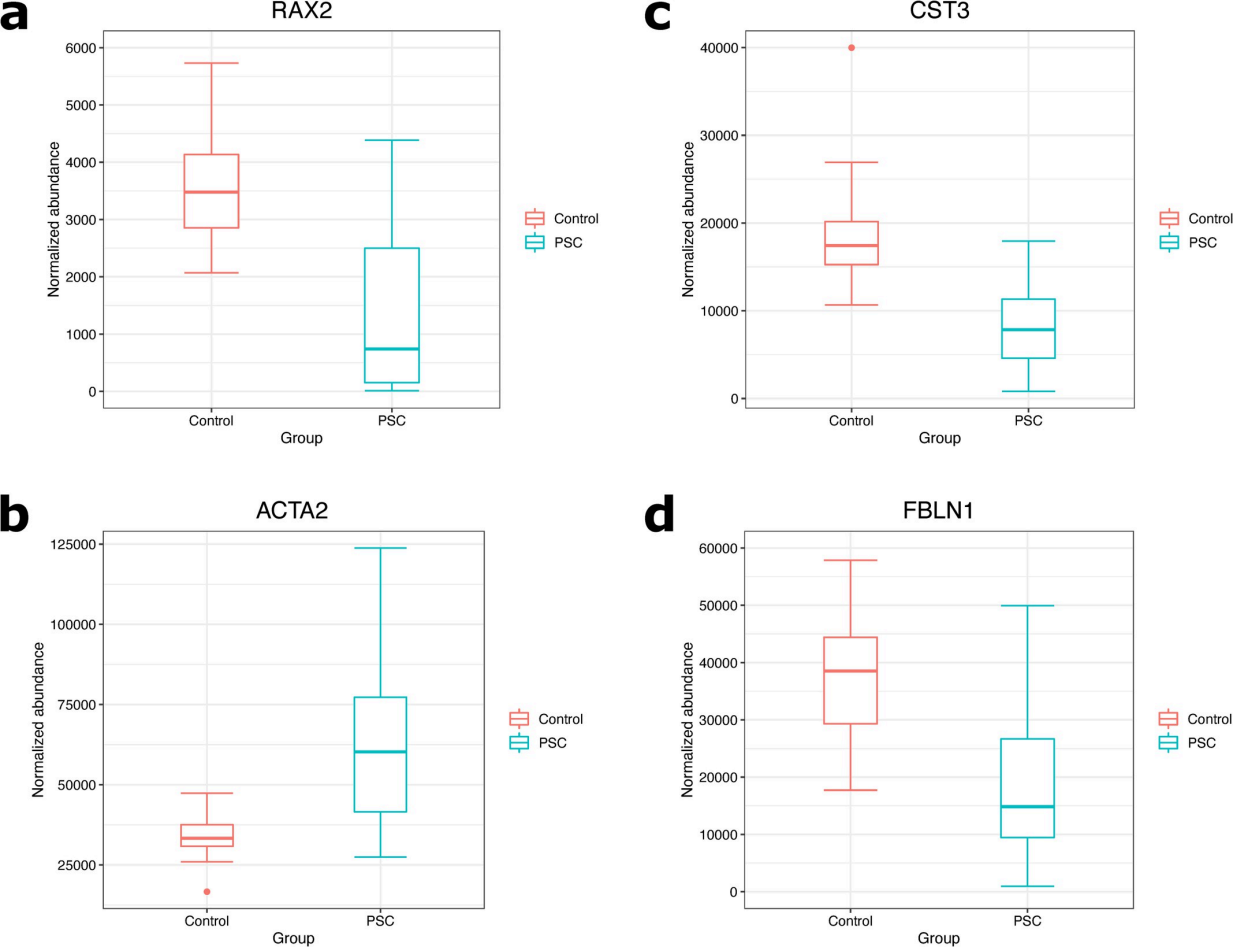
Box plots showing the normalized abundances of the four serum proteins with the highest AUC values in non-PSC controls and PSC patients. A) The box plot for retina and anterior neural fold homeobox protein 2. B) The box plot for for cystatin-C. C) The box plot for actin, aortic smooth muscle. D) The box plot for fibulin-1.

When serum samples from PSC patients and non-PSC controls were compared, a total of 112 proteins passed the cutoff of a Mann-Whitney U test p-value of less than 0.05 and were considered to have significantly different levels between the groups. The top 20 proteins according to fold change are given in Table 2 and all 112 proteins can be found marked in blue in S3 Table. The protein with the largest fold change (2.8) was biliverdin reductase A, which displayed higher levels in PSC patients. ROC analysis was performed for these 20 proteins and the results are also presented in Table 2. Multiple serum proteins were found to have AUC values of >0,8. The proteins with the highest AUC values were cystatin-C (0,942), retina and anterior neural fold homeobox protein 2 (0,900), actin, aortic smooth muscle (0,895), and fibulin-1 (0,883). The ROC curves for these four proteins are shown in Fig 2 and box plots in Fig 3.

**Table 2:** The top 20 proteins according to fold change that passed the cutoff of a Mann-Whitney p-value of less than 0.05 when serum samples from PSC patients and non-PSC controls were compared.

Accession	Peptide count	Unique peptides	Confidence score	Mann-Whitney P-value	Power	Max fold change (PSC/control)	Protein name	Gene name	AUC	AUC 95% CI	
										Lower	Upper
P53004	6	2	28,5	1,81E-03	0,945	2,8	Biliverdin reductase A	BLVRA	0,721	0,601	0,831
Q9Y6X9	2	2	8,4	5,15E-05	1,000	2,7	MORC family CW-type zinc finger protein 2	MORC2	0,795	0,683	0,890
Q96IS3	2	2	6,9	6,62E-08	1,000	-2,7	Retina and anterior neural fold homeobox protein 2	RAX2	0,900	0,830	0,958
P01034	7	5	34,9	2,73E-09	1,000	-2,3	Cystatin-C	CST3	0,942	0,885	0,985
P10721	7	2	36,5	1,18E-05	0,996	-2,3	Mast/stem cell growth factor receptor Kit	KIT	0,821	0,738	0,896
Q92496	5	4	30,1	9,33E-03	0,974	2,2	Complement factor H-related protein 4	CFHR4	0,679	0,531	0,814
A6NEY8	4	2	22,5	1,01E-04	0,999	2,2	Putative prolyl-tRNA synthetase associated domain-containing protein 1	PRORSD1P	0,782	0,680	0,874
P23142	5	3	29,1	2,29E-07	1,000	-2,1	Fibulin-1	FBLN1	0,883	0,805	0,944
P61626	3	3	20,6	2,36E-05	1,000	-1,9	Lysozyme C	LYZ	0,809	0,707	0,895
Q8TCB7	3	2	24,3	3,36E-04	0,999	1,9	Methyltransferase-like protein 6	METTL6	0,758	0,645	0,861
Q9P2M7	2	2	9,3	2,39E-03	0,998	1,9	Cingulin	CGN	0,714	0,564	0,850
Q9NP55	2	2	12,6	9,80E-03	0,579	-1,8	BPI fold-containing family A member 1	BPIFA1	0,677	0,534	0,802
Q96A32	3	2	17,7	8,50E-06	1,000	1,8	Myosin regulatory light chain 2_ skeletal muscle isoform	MYLPF	0,826	0,736	0,903
P62736;P63267;P68032;P68133	5	4	30,4	9,90E-08	1,000	1,8	Actin_ aortic smooth muscle	ACTA2	0,895	0,823	0,952
O75037	3	2	18,6	1,40E-05	0,996	-1,7	Kinesin-like protein KIF21B	KIF21B	0,818	0,702	0,914
Q9Y5Y7	2	2	11,3	3,92E-03	0,996	1,6	Lymphatic vessel endothelial hyaluronic acid receptor 1	LYVE1	0,702	0,577	0,816
Q969I3	4	2	31,0	7,17E-04	0,994	-1,6	Glycine N-acyltransferase-like protein 1	GLYATL1	0,742	0,632	0,842
O94910;Q9C0H6;Q9H5F2	3	2	17,3	1,78E-05	0,933	-1,6	Adhesion G protein-coupled receptor L1	ADGRL1	0,814	0,725	0,895
P35908	10	3	75,0	4,39E-04	0,998	1,5	Keratin_ type II cytoskeletal 2 epidermal	KRT2	0,752	0,598	0,881
Q9Y6D0	2	2	12,5	3,32E-03	0,958	-1,5	Selenoprotein K	SELENOK	0,706	0,598	0,805

The proteins that passed this cutoff and also have a fold change of >2 (and are therefore considered biologically relevant) are shaded in blue.

#### Pathway analysis

**Fig 4:**
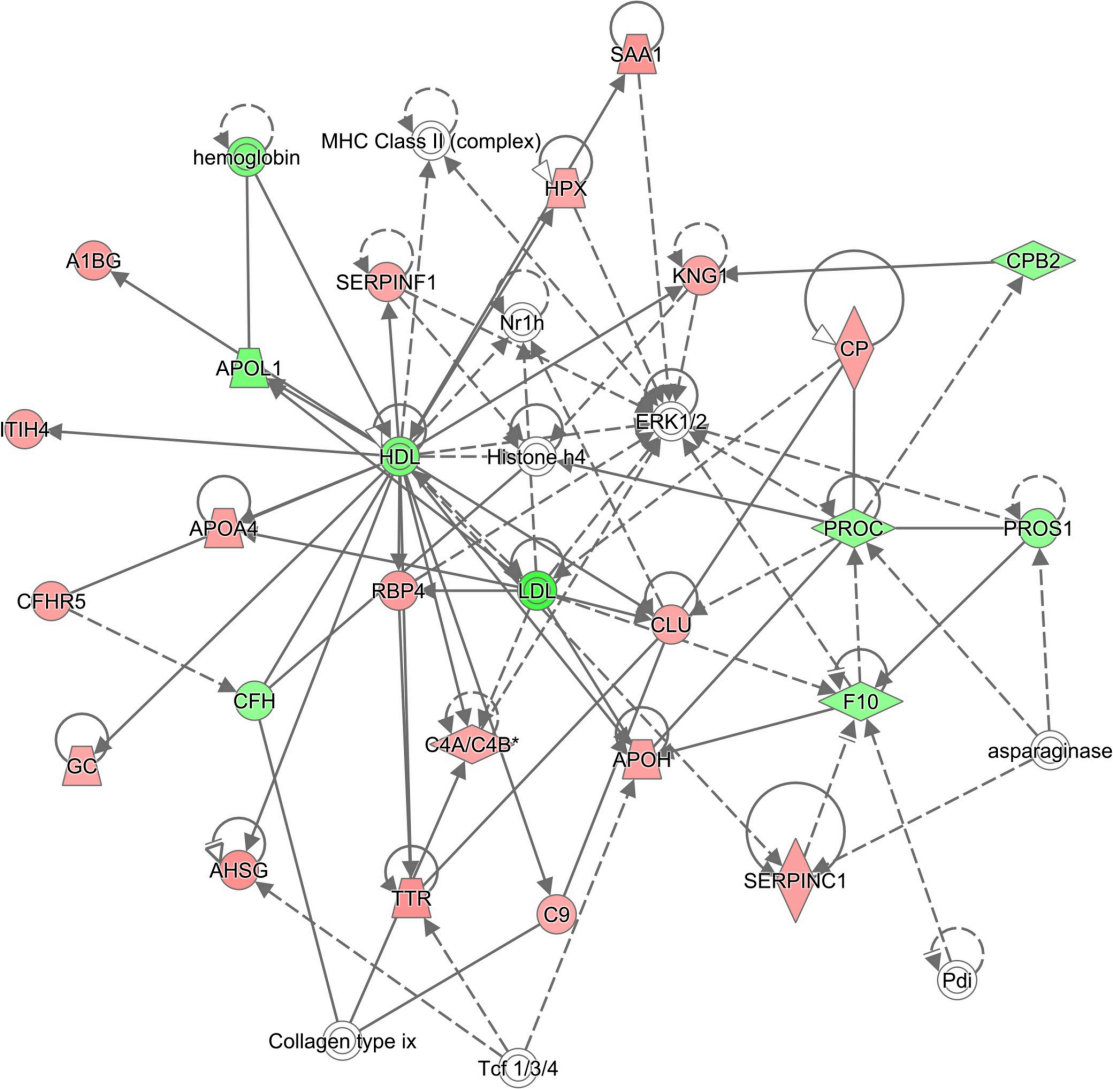
The top network of protein-protein interactions generated by IPA when serum samples from PSC patients and controls were compared. Proteins in red are proteins that were identified in this study and which displayed higher levels in PSC patients. Proteins in green are proteins that displayed lower levels in PSC patients. Proteins in white are proteins that, although not identified in this study, play roles in this network and interact with the proteins identified.

Pathway analysis was performed using IPA and discovered multiple canonical pathways that were enriched in the serum protein dataset. The top five pathways enriched were LXR/RXR Activation, FXR/RXR Activation, Acute Phase Response Signaling, Coagulation System, and Intrinsic Prothrombin Activation Pathway. All canonical pathways enriched are given in S2 Fig. Networks of protein-protein interactions were also obtained during pathway analysis. The top network is shown in Fig 4 and was found to be associated with the following functions: Protein Synthesis, Hematological System Development and Function, and Organismal Functions. Only proteins that passed the cutoff of a Mann-Whitney p-value of less than 0.05 were used for pathway analysis.

### Comparison of bile and serum proteomics of PSC patients and non-PSC controls

Several of the serum proteins with significantly different levels between the two groups also had significantly different levels when bile samples were analyzed (S2 and S3 Tables). These proteins included corticosteroid-binding globulin, clusterin, and CD5 antigen-like, which all had significantly higher levels in the serum and bile samples from PSC patients. Corticosteroid-binding globulin had a fold change of 1.2 in serum and 2.1 in bile, while clusterin had a fold change of 1.2 in serum and 4.3 in bile. CD5 antigen-like had a fold change of 1.03 in serum but 4.9 in bile.

## Discussion

In this study, we have compared the bile and serum proteomic profiles of PSC patients and non-PSC controls. We discovered 64 proteins that had significantly different levels in bile samples and 112 proteins that had significantly different levels in serum samples that were compared between the two groups. The differentially expressed proteins identified in bile samples had much larger fold changes, up to 129, than those identified in serum samples. The proteins identified in bile samples could be more specific markers for PSC than those identified in serum samples, although bile samples are difficult to obtain due to the invasiveness of sample collection. Bile samples are therefore unsuitable for the screening of PSC but could be aid in the differential diagnosis of PSC. The use of serum proteins as biomarkers is therefore ideal due to the minimally invasive nature of sample collection and these proteins could be used as a first step for the screening and diagnosis of PSC. ROC analysis was therefore performed on the top 20 (according to fold change) Mann-Whitney U test-passing serum proteins (Table 2). Cystatin-C was found to have an AUC value of 0.942 and retina and anterior neural fold homeobox protein 2 an AUC value of 0.900, making them outstanding classifiers for PSC vs. no PSC.

Pathway analysis revealed LXR/RXR Activation and FXR/RXR Activation to be among the top five canonical pathways enriched in both the bile and serum datasets (S1 and S2 Figs). FXR and RXR are nuclear receptors that are involved in normal bile homeostasis and respond to cholestasis [1]. FXR is also involved in the regulation of bile acid synthesis, transport, and detoxification and forms a heterodimer with RXR [22]. LXR is also a nuclear receptor that forms obligate heterodimers with RXR and acts as a cholesterol sensor. LXR regulates the metabolism of cholesterol and bile acids and has an important role in the regulation of bile acid synthesis [23, 24]. Targeting FXR using FXR agonists may mitigate the cholestasis that is a hallmark of PSC. A study by Trauner et al. found that use of the FXR agonist cilofexor improved markers of cholestasis and led to reductions in serum ALP in PSC patients [25]. Our findings that LXR/RXR Activation and FXR/RXR Activation were enriched in our dataset confirm the cholestasis and alterations in bile metabolism seen in PSC and indicate that we have correctly identified proteins whose levels differ between PSC patients and non-PSC controls.

Although the bile and serum proteomes were very different from each other when samples from PSC patients and non-PSC controls were compared, we did identify three proteins (corticosteroid-binding globulin, clusterin, and CD5 antigen-like) with significantly higher levels in both bile and serum samples from PSC patients. Very little is known about the role of these proteins in the pathogenesis and progression of PSC, although a study by Aigelsreiter et al. showed that clusterin was present in bile samples from PSC patients. The authors proposed that clusterin may act as an extracellular chaperone that could either protect bile duct epithelia from damage or biliary proteins from misfolding [26]. The fold changes of these three proteins were higher in bile than serum (S2 and S3 Tables), which indicates that the bile samples studied serve as a positive control for the presence of PSC. Given that the levels of these proteins were significantly altered in the proteomic profiles of both bile and serum, they could also be of value as new serum markers for the differential diagnosis of PSC. However, despite the statistical significance, the fold changes of these three proteins were <2 in serum when compared between PSC patients and controls and were therefore not considered biologically relevant. Further studies and validation are needed to investigate their potential as biomarker candidates.

One strength of this pilot study is the large patient cohort, including both non-advanced, early-stage disease and advanced bile duct disease, and the representative number of bile samples analyzed. As bile samples require ERC to be performed in order to be collected and are therefore difficult to obtain, there are few studies of bile proteomics and fewer studies that have analyzed and compared both the bile and serum proteomes in PSC patients. Here, we have analyzed bile and serum samples from the same 80 PSC patients, allowing us to compare the two proteomes and their alterations. Further, the patient cohort included in this study was well-characterized, and the PSC patients included in this study were confirmed not to have dysplasia or CCA. This enabled us to identify proteins that could be of value specifically in screening for and diagnosing PSC, a premalignant disease, which could enable earlier intervention before disease progression to dysplasia and CCA.

Due to the invasive nature and technical difficulties of obtaining bile samples, only six non-PSC patients were used as controls, leading to imbalanced groups. Both bile and serum samples for the control cohorts were obtained from patients with suspected PSC who underwent ERC and follow-up for two years and who were subsequently not diagnosed with PSC. As only patients with suspected PSC underwent ERC, this also limited the number of patients in the control cohorts, an issue that we are aware of but that could not be avoided at the time this study was performed. In this study, we used the Mann-Whitney U test to analyze the differences between the groups. Unequal sample size is not an issue for the Mann-Whitney U test, although unequal sample size can decrease power. The power of the identified proteins with a Mann-Whitney U test p-value of <0.05 and a fold change of >2 generally have a high power of >0.9, indicating that the differences in their levels between PSC patients and non-PSC controls are truly significant. The control cohorts represent a heterogeneous group of other liver diseases, allowing us to identify markers for the differential diagnosis of PSC, something of great importance due to the increased risk of CCA in PSC patients. We have continued to collect bile and serum samples from non-PSC patients and aim to compare the proteomes of healthy controls, non-PSC patients, PSC patients, and CCA patients in a future study that will also include more balanced groups.

In this study, we show that PSC significantly alters both the bile and serum proteome of PSC patients. The bile proteome of PSC patients has previously been shown to display disease-specific changes [27]. However, the bile and serum proteomes of the same PSC patients have not been previously compared to non-PSC controls. Here, we have identified multiple serum proteins (including three proteins that displayed significantly higher levels in both bile and serum samples from PSC patients) that could be of value as new markers for the screening and differential diagnosis of PSC. As their levels can be measured in serum samples, this would provide a way to easily screen for PSC and distinguish it from other cholestatic liver diseases, a significant improvement over cholangiography. This could subsequently help diagnose PSC at an early stage, which would allow patients to be monitored closely for possible malignant changes and enable intervention at an earlier stage. Further validation of the clinical utility of the serum proteins identified is needed, as are analyses to identify additional putative biomarkers or their combinations that could be used for the screening and differential diagnosis of PSC at an early stage. In conclusion, we propose several proteins that could be valuable as candidates for new non-invasive biomarkers in this pilot study, although additional research is needed.

## Supporting information

S1 Fig All canonical pathways enriched when bile samples from PSC patients and non-PSC controls were compared using Ingenuity Pathway Analysis. (TIFF)

S2 Fig All canonical pathways enriched when serum samples from PSC patients and non-PSC controls were compared. (TIFF)

S1 Table Information about the patients included in this study. (XLSX)

S2 Table All 301 proteins with two or more unique peptides quantified in bile samples from PSC patients and non-PSC controls. Proteins that passed the cutoff of a Mann-Whitney p-value of less than 0.05 are shaded in blue. Significantly different proteins that also have a fold change of >2 are further given in bold.
(XLSX)

S3 Table All 207 proteins with two or more unique peptides quantified in serum samples from PSC patients and non-PSC controls. Proteins that passed the cutoff of a Mann-Whitney p-value of less than 0.05 are shaded in blue. Significantly different proteins that also have a fold change of >2 are further given in bold.
(XLSX)

## Abbreviations

ALP: Alkaline phosphatase
AUC: Area under the curve
CCA: Cholangiocarcinoma
ERC: Endoscopic retrograde cholangiography
IBD: Inflammatory bowel disease
IPA: Ingenuity Pathway Analysis
LXR: Liver X receptor
PSC: Primary sclerosing cholangitis
ROC: Receiver operating characteristic
RXR: Retinoid X receptor
SSC: Secondary sclerosing cholangitis
UPLC-UDMS^E^: Ultra performance liquid chromatography-ultra definition mass spectrometry
