# Supplementary figures and images for: Quantitative bile and serum proteomics for the screening and differential diagnosis of primary sclerosing cholangitis
Source: PLoS One. 2022 Aug 25;17(8):e0272810. doi: 10.1371/journal.pone.0272810 (PMC9409575; doi:10.1371/journal.pone.0272810)

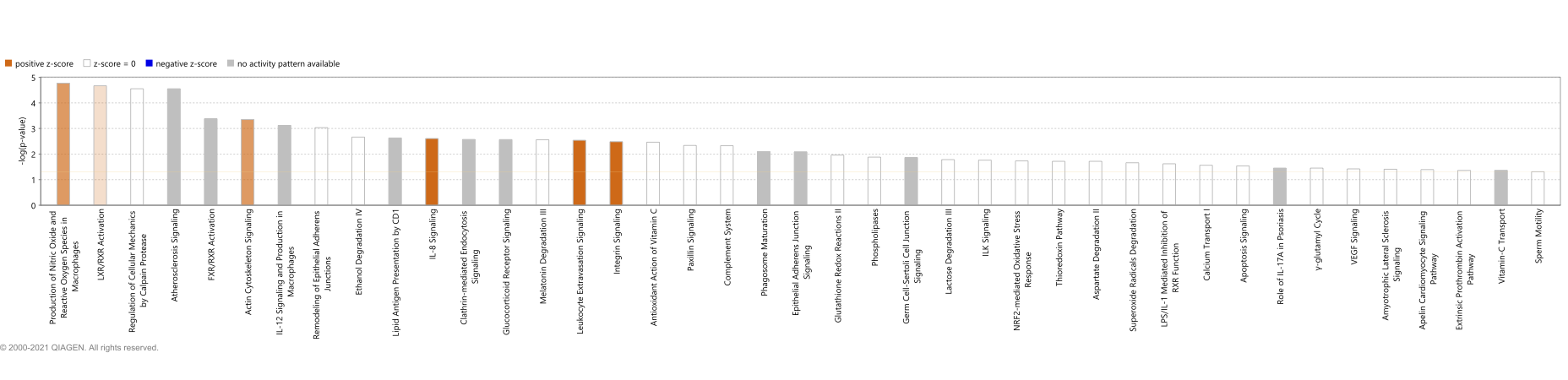

Supplement: S1 Fig — (TIFF) [file pone.0272810.s001.tiff]

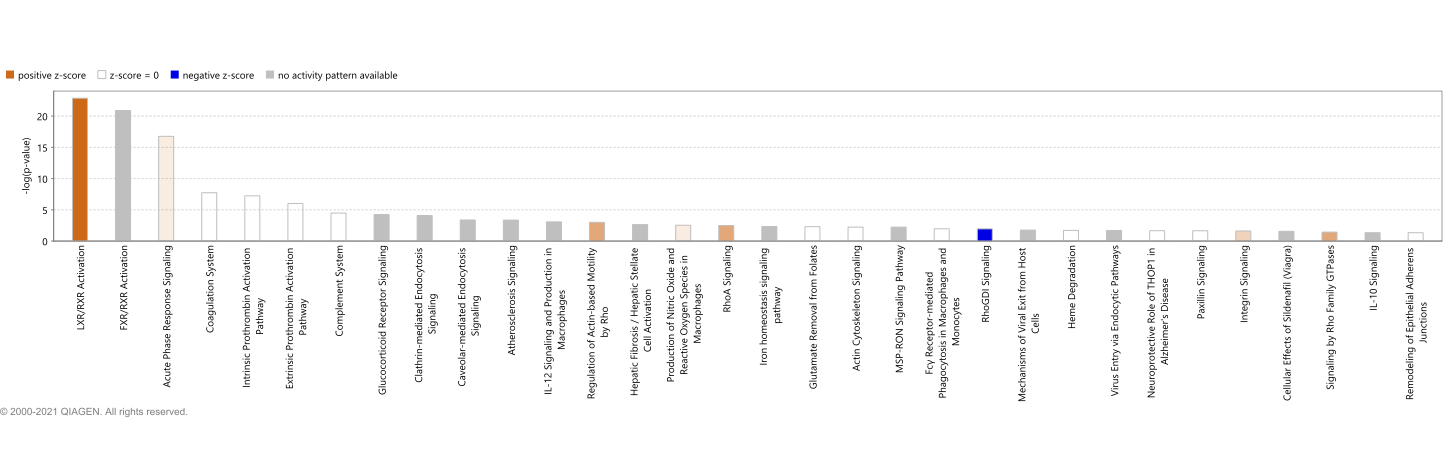

Supplement: S2 Fig — (TIFF) [file pone.0272810.s002.tiff]
